# When Bacteria and Viruses Collide: A Tale of *Chlamydia trachomatis* and Sexually Transmitted Viruses

**DOI:** 10.3390/v15091954

**Published:** 2023-09-19

**Authors:** Ehsan Ghasemian, Emma Harding-Esch, David Mabey, Martin J. Holland

**Affiliations:** Department of Clinical Research, London School of Hygiene & Tropical Medicine, London WC1E 7HT, UK; emma.harding-esch@lshtm.ac.uk (E.H.-E.); david.mabey@lshtm.ac.uk (D.M.); martin.holland@lshtm.ac.uk (M.J.H.)

**Keywords:** sexually transmitted infection, *Chlamydia trachomatis*, human immunodeficiency virus, human papillomavirus, herpes simplex virus, co-infection

## Abstract

The global incidence of sexually transmitted infections (STIs) remains high, with the World Health Organization (WHO) estimating that over 1 million people acquire STIs daily. STIs can lead to infertility, pregnancy complications, and cancers. Co-infections with multiple pathogens are prevalent among individuals with an STI and can lead to heightened infectivity and more severe clinical manifestations. *Chlamydia trachomatis* (CT) is the most reported bacterial STI worldwide in both men and women, and several studies have demonstrated co-infection of CT with viral and other bacterial STIs. CT is a gram-negative bacterium with a unique biphasic developmental cycle including infectious extracellular elementary bodies (EBs) and metabolically active intracellular reticulate bodies (RBs). The intracellular form of this organism, RBs, has evolved mechanisms to persist for long periods within host epithelial cells in a viable but non-cultivable state. The co-infections of CT with the most frequently reported sexually transmitted viruses: human immunodeficiency virus (HIV), human papillomavirus (HPV), and herpes simplex virus (HSV) have been investigated through in vitro and in vivo studies. These research studies have made significant strides in unraveling the intricate interactions between CT, these viral STIs, and their eukaryotic host. In this review, we present an overview of the epidemiology of these co-infections, while specifically delineating the underlying mechanisms by which CT influences the transmission and infection dynamics of HIV and HSV. Furthermore, we explore the intricate relationship between CT and HPV infection, with a particular emphasis on the heightened risk of cervical cancer. By consolidating the current body of knowledge, we provide valuable insights into the complex dynamics and implications of co-infection involving CT and sexually transmitted viruses.

## 1. Introduction

Sexually transmitted infections (STIs) result in serious reproductive and sexual health sequelae, including infertility, pregnancy complications, and the development of cancers [1,2]. In 2018, the direct medical costs of new STI cases in the United States of America (USA) alone amounted to approximately $16 billion [3]. A high proportion of patients with STIs are co-infected with multiple pathogens [4,5,6,7,8]. Sexually transmitted co-infections are common because the pathogens share transmission routes and the majority of infections are asymptomatic, which results in patients not seeking treatment [8,9]. High-risk sexual behavior including having sex with multiple partners, inconsistent condom use, and belonging to specific sexual networks such as men who have sex with men (MSM) are associated with increased risk of co-infection with multiple pathogens [10,11,12,13]. Other factors associated with co-infection include younger age, having a history of previous STIs, and low educational status [14,15,16].

The simultaneous presence of sexually transmitted pathogens in co-infections can result in heightened infectivity, exacerbated symptoms, increased odds of pathogen transmission, and reduced effectiveness of treatment and prevention interventions [8,9,17,18,19]. Previous findings indicate that the concordance of sexually transmitted pathogens within sexual partnerships is poor, and the limited concordance of infection is exacerbated by the presence of a single infection in one person and co-infection with multiple pathogens in the other [20,21,22,23,24].

Globally, *Chlamydia trachomatis* (CT) is the most reported bacterial STI [25,26,27,28]. CT is an obligate intracellular bacterium that infects epithelial cells at mucosal surfaces [29,30,31,32]. The ability of CT to infect the urogenital and rectal tracts in the absence of significant local or systemic symptoms allows the pathogen to go untreated for extended periods [33]. Previous studies have demonstrated the ability of CT to facilitate the infection of sexually transmitted viruses [34,35,36,37,38,39]. For instance, CT enhances human immunodeficiency virus (HIV) infection by increasing the expression of HIV-1 primary and co-receptors on epithelial cells, which decreases epithelial integrity and enhances cell-associated virus migration across the epithelial barrier. CT also enhances the accumulation of CD4+ T cells that express the HIV co-receptors CXCR4 and CCR5 at the site of infection [38,39,40,41]. Among different sexually transmitted viruses, co-infections of HIV, human papillomavirus (HPV), and herpes simplex virus (HSV) with CT have repeatedly been reported [42,43,44,45,46,47,48], and several in vitro and in vivo studies have attempted to unveil the interaction of these viral STIs with CT and their eukaryotic host [18,39,49,50,51,52,53].

The aim of this review is to examine the available data on the mechanisms through which CT influences the transmission and infection of HIV and HSV, and in the case of HPV infection, the risk of cervical cancer. We begin with a concise introduction to CT biology and pathogenesis, followed by a summary of each co-infection. Each section will provide (i) a brief overview of the infection and immunopathology associated with the respective virus, (ii) the epidemiology of the co-infections, (iii) the interaction of CT and sexually transmitted viruses with each other and with their host, and (iv) a conclusion. We do not discuss the impact of CT infection on the vaginal microbiome, which in itself may be associated with bacterial vaginosis facilitating secondary STIs [36,54,55,56,57]. Instead, we focus on the direct interaction of CT with viral STIs. While co-infections of CT with other sexually transmitted bacteria, such as *Neisseria gonorrhoeae* (NG) and *Mycoplasma genitalium*, are common [58,59,60,61], addressing them lies beyond the scope of this review.

## 2. *Chlamydia trachomatis* Biology and Pathogenesis

There are distinct CT genovars that target specific anatomical sites of the human body. CT genovars A-C infect the eyes, resulting in a debilitating disease known as trachoma [62], CT genovars D-K, on the other hand, target the urogenital tract and have the potential to cause pelvic inflammatory disease (PID), tubal factor infertility, and ectopic pregnancy [25,63]. Lastly, CT genovars L1-L3 specifically cause lymphogranuloma venereum (LGV), which is an ulcerative disease that affects the anogenital area [64].

CT has a biphasic developmental cycle [29]. The extracellular elementary body (EB) is the infectious form and is characterized as being metabolically inert [65]. Upon host cell contact, the EBs bind to heparan sulfate proteoglycans on epithelial cells, followed by interaction with a variety of cell surface receptors such as the mannose receptor, mannose-6-phosphate receptor, epidermal growth factor receptor, fibroblast growth factor receptor, platelet-derived growth factor receptor, ephrin receptor A2, protein disulfide isomerase, and β1 integrin [65,66]. CT attachment to the host cell is followed by an active actin remodeling process that facilitates entry of the microorganism into the cytoplasm [66,67]. Following entry, within a parasitophorous vacuole termed the inclusion, the EBs transform into the noninfectious replicative form of the organism called the reticulate body (RB) [68,69]. RBs hijack cellular machinery to utilize nutrients within the host cytoplasm and promote bacterial replication by binary fission [65]. RBs’ differentiation back into EBs coincides with a decreasing pool of nutrients and adenosine triphosphate [65]. Finally, EBs exit the cell through host cell lysis or extrusion of the cytoplasmic inclusion [70]. Under unfavorable growth conditions characterized by the presence of antibiotic or host immune responses, a state of persistence within the inclusion begins in which CT remains viable but atypical, with an enlarged, aberrant form and quiescent metabolism [65,66]. In this persistent form, CT substantially upregulates the synthesis and release of its chaperonins (Hsp60s) known as GroEL from the host cells [71,72,73]. GroEL acts as a virulence factor that promotes the survival of the bacterium by modulating the host immune responses and suppressing the activation of pro-inflammatory responses, inhibiting apoptosis, and assisting in nutrient acquisition and utilization. Additionally, GroEL triggers immune responses in the host, contributing to the pathogenicity of the infection [71,72].

Upon infection, a local pro-inflammatory response to CT infection initiated by host epithelial cells recruits immune cells such as macrophages, neutrophils, dendritic cells (DCs), natural killer (NK) cells, T cells, and B cells to the site of infection [65,66,74]. Immune cells secrete pro-inflammatory cytokines and chemokines and stimulate a chronic inflammatory environment through the release of reactive oxygen species (ROS) and degradative enzymes including defensins, elastase, collagenase, cathepsins, and lysozyme [75]. CT deploys different mechanisms to evade the host immune responses, including the CT-directed production of multiple proteases, the inhibition of major histocompatibility complex (MHC) expression by induction of beta interferon (IFN-β), and the inhibition of apoptosis in CT-infected cells [65]. Among different CT proteases, the chlamydial protease-like activity factor (CPAF) is important for the development of CT [65,76,77,78,79]. There are studies showing that CPAF can directly suppress the release of pro-inflammatory cytokines, such as CXCL10, and degrade innate immune effectors such as the nuclear factor-kappa B (NF-κB) as well as other transcription factors that initiate the production of multiple pro-inflammatory mediators [65,80,81]. CPAF degrades the transcription factors RFX5 and USF-1, resulting in the inhibition of the expression of MHC class I and II molecules [65,82]. Moreover, released CPAF from the infected host epithelial cells cleaves formyl peptide receptor 2 (FPR2), which is a natural activator of polymorphic nuclear leukocytes (PMNs) located on the surface of neutrophils [83,84]. As a consequence, affected neutrophils are paralyzed and fail to respond to activation by different stimuli, highlighting CPAF-mediated inactivation of PMNs as a crucial event in asymptomatic CT infection [83,84]. Therefore, it is possible to envision how an organism that manipulates the local immune responses might also suppress or alter the host response to other pathogens that are present at the same time and increase the likelihood of secondary pathogen infection [38].

## 3. Co-Infection of *Chlamydia trachomatis* and Human Immunodeficiency Virus

### 3.1. Infection and Immunopathology of Human Immunodeficiency Virus

HIV-1 and HIV-2 belong to the Retroviridae family and are classified as single-stranded RNA viruses (63). Globally, HIV-1 is responsible for the majority of HIV infections [85,86]. According to WHO, 630,000 out of 39 million people who were living with HIV globally by the end of 2022 died from HIV-related causes [87]. The infection is a dynamic process with different rates of disease progression, and clinically it causes progressive impairment of immune function [88,89].

The first contact sites between HIV and the host are mucosal epithelia, which play a critical role in determining the success of establishing a systemic infection [90]. Schust et al. [38] reported that epithelial cells are the first cells encountered by luminal HIV in the endocervix and, therefore, play a central role in viral transmission. The internalization of the virus into epithelial cells occurs by multiple entry pathways, including clathrin-, caveolin/lipid raft-associated endocytosis, and micropinocytosis [90]. HIV enters lymphocytes and monocytes through cognate recognition of the viral glycoprotein gp120 with the cell surface CD4 molecule [90,91]. After binding to CD4, HIV also interacts with specific co-receptors on the cell surface [90,91]. The co-receptors most commonly involved are CCR5 (C-C chemokine receptor type 5) and CXCR4 (C-X-C chemokine receptor type 4) [90,92,93]. There are three common combinations of co-receptor usage and cellular tropism: (i) CCR5-utilizing T-cell-tropic (T-tropic) HIV, which accounts for transmitted/founder viruses and most circulating viruses, (ii) CXCR4-utilizing T-tropic HIV, arising in approximately 50% of late-stage HIV patients, and (iii) macrophage-tropic (M-tropic) HIV that principally uses CCR5 co-receptors, and arises in some individuals late in infection and in T-cell-depleted environments such as in advanced HIV-1 disease [92,93]. The interaction of these proteins induces the binding of the viral gp41 to heparan sulfate on the host plasma membrane [94,95]. This triggers the fusion of the viral envelope and the release of the capsid into the cytoplasm [94,95].

HIV infection induces a profound immune dysfunction, with abnormalities in cellular and humoral immune responses, including (i) CD4 lymphopenia, (ii) neutropenia, (iii) decreased NK cell-mediated cytotoxicity, (iv) decreased phagocytosis, chemotaxis, intracellular killing, and cytokine expression in monocytes, (v) decreased B cell number, and (vi) loss of specific antibody response [90,91]. Several studies have shown that the host and virus jointly determine disease progression after the infection, in which early events in the acquisition, replication, and transmission of the viral particles and the activation level of innate immunity are critical [39,91]. Without treatment, HIV-1-positive individuals often die of acquired immune deficiency syndrome (AIDS) several years after infection [89].

### 3.2. Epidemiology of Chlamydia trachomatis and Human Immunodeficiency Virus Co-Infections

Among HIV-positive cases, a notable portion (7%) are infected with CT [96]. Studies have reported a higher sensitivity to HIV infection in CT-positive individuals compared with non-infected controls [97,98]. Out of 46 patients diagnosed with HIV-STI co-infection in India, CT was the most prevalent STI affecting 20 (43.5%) patients [99]. Among 234 HIV patients diagnosed with HIV in Georgia, the seroprevalence of CT was 23.93% [42]. A study in rural Tanzania showed that improving the availability of effective treatment of CT and other bacterial STIs reduced the incidence of HIV infection by about 40% [100]. Moreover, CT-infected individuals have been shown to be more likely to have higher levels of HIV and genital shedding of the virus, and subsequently higher chances of HIV transmission, than those infected with HIV alone [101,102,103]. Increased risk of HIV infection is particularly prominent among men compared with those without rectal CT infection [104,105,106,107]. Bernstein et al. [105] showed that in MSM with a history of rectal NG or CT infections, the risk of HIV acquisition increased by eightfold compared with those without a history of CT or NG infection [105]. A study in Washington State, USA, involving 6577 HIV-negative MSM unveiled that the incidence of HIV infection among those with rectal CT was 1.6 per 100 person-years, whereas the estimated incidence among all MSM in the state was 0.4 [104]. Several studies have shown that treatment of CT is associated with significant reductions of HIV-1 RNA copies in genital secretions [98,102,106,108]. Median HIV-1 RNA copies in cervical secretions and seminal fluid were markedly diminished upon successful resolution of bacterial infections among women with cervicovaginal NG or CT in Côte d’Ivoire and Kenya, as well as men with gonorrheal or chlamydial urethritis in the United Kingdom [103,108,109].

### 3.3. Interaction of Chlamydia trachomatis with Human Immunodeficiency Virus in Co-Infections

In general, higher odds of HIV transmission among CT-infected individuals can be explained by a higher viral load due to a greater number of HIV-infected immune cells in genital secretions induced by CT-related inflammatory responses. Prior studies have demonstrated that an existing CT infection in the cervical epithelium provides additional targets for the virus by recruiting immune cells to the site of infection [34,110,111,112]. Active infection of the endocervix with CT results in a local accumulation of CD4+ T cells that express the HIV co-receptors [113]. Schust et al. [38] showed that CT infection led to more than a log-fold increase in the number of CD4+ cells collected from the endocervix, of which the majority expressed CXCR4 and/or CCR5. Moreover, after CT infection, antigen-presenting cells (APCs), such as macrophages, are recruited to the site of infection [89]. Macrophages are one of the major cellular reservoirs for latent HIV-1 infection and contribute to early-stage virus transmission and dissemination throughout the host [114]. A study involving MSM with asymptomatic CT and NG infection reported increased systemic immune activation, a cofactor for increased susceptibility to HIV-1 infection, due to a higher frequency of CD8+ T cells co-expressing activation markers (HLA-DR and CD38), an exhaustion marker (PD-1), and senescence markers (CD57 and CD28) [115].

Prior studies have shown that the columnar epithelium of the endocervix in women is a primary site of infection for CT and a permissive site for HIV entry [39,96,102]. Infection with CT may decrease epithelial integrity and transepithelial resistance, and increase paracellular permeability [38,40,41]. This could facilitate paracellular migration of HIV, allowing direct contact with infiltrating immune cells [39]. Buckner et al. [39], using endocervical epithelial cells, showed that CT infection enhanced cell-associated virus migration across the epithelial barrier.

Finally, mechanisms initiated by epithelial cells could directly be involved in the enhancement of HIV replication in target cells [38,90,113]. Cohen et al. [116] showed that HIV-1 RNA concentration in the seminal plasma of men with urethritis was eight times higher than those without urethritis, despite similar CD4 T-cell counts. A further in vitro study using epithelial cells showed CT infection increases cell surface expression of HIV-1 primary and co-receptors [38]. Compared with un-infected epithelial cells, those infected with CT significantly increased the cell surface expression of galactosylceramide, an HIV-1 alternative primary receptor, and the most commonly studied HIV-1 co-receptors CXCR4 and CCR5 [38,39,90]. CT infection enhanced HIV-1 binding to epithelial cells and, subsequently, increased virus levels in co-cultures of HIV-exposed epithelial cells and susceptible CD4+ lymphocytes [39]. Basolateral supernatants from CT-infected epithelial cells enhanced HIV infection in exposed peripheral blood mononuclear cells (PBMCs) and CD4+, CCR5+ cells [39]. Buckner et al. [39] suggested that IL-1α, or an unidentified factor, or a combination of factors present in the basolateral supernatants from CT-infected epithelial cells, may be acting upon target cells to facilitate HIV entry and/or replication. Ex vivo infection of HIV in PBMCs from healthy donors and those infected with CT showed significantly higher viral levels in those with CT infection [117,118]. Similarly, CT stimulation of PBMCs isolated from healthy donors showed enhanced susceptibility to HIV-1 [118]. Stimulating CD4 T cells with CT led to viral release and the upregulation of CCL3L1/CCL3L3, a paralog of CCL3, harboring more HIV-1 copies in CD4 T cells, while causing inhibition of HIV-1 through interaction with the CCR5 co-receptor on the cell membrane [118]. Notably, the expression threshold of CCL3L1 needed to cause significant inhibition of the CCR5 co-receptor on the cell surface could not be achieved by CT stimulation of the cells [118].

**Figure 1:**
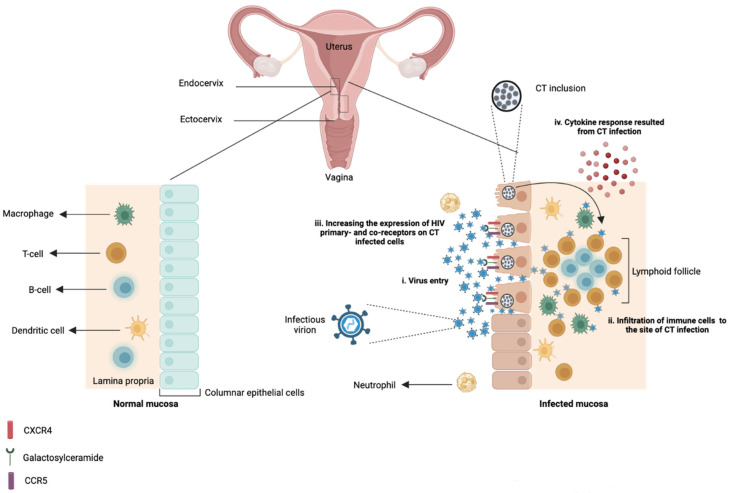
Interplay of *Chlamydia trachomatis* and host immune responses, and its impact on human immunodeficiency virus infection in the endocervix. The columnar epithelium of the endocervix in women is a primary site of infection for *Chlamydia trachomatis* (CT) and a permissive site for human immunodeficiency virus (HIV) entry. Infection of CT in the columnar epithelial cells of the cervix induces pro-inflammatory responses that can lead to an influx of macrophages and neutrophils and the formation of lymphoid follicles in the submucosa. These lymphoid follicles contain B cells and T cells. CT infection in endocervical epithelial cells could facilitate HIV infection by (i) decreasing epithelial integrity and transepithelial resistance and enhancing paracellular permeability by providing direct contact with underlying infiltrating immune cells, (ii) providing additional targets for the virus by recruiting immune cells such as CD4+ T cells and macrophages to the site of infection, (iii) increasing cell surface expression of HIV-1 alternative primary receptor, galactosylceramide, and co-receptors, CXCR4 and CCR5 on CT-infected epithelial cells, (iv) secretion of cytokines/unidentified factors that may be acting directly upon CD4+ cells to facilitate HIV entry and/or replication. Created with BioRender.com.

Figure 1 depicts the interaction of CT with HIV during the course of a co-infection in the endocervix.

### 3.4. Conclusions

These findings suggest that CT infection of endocervical epithelial cells could facilitate HIV access to underlying susceptible cell types, the establishment of a founder virus population, and, ultimately, a higher probability of acquisition and transmission of the virus. Although the efficiency of the sexual transmission of HIV is poor, perhaps as infrequently as 1 in every 1000 episodes of sexual intercourse, STIs such as CT can increase the efficiency of HIV transmission by increasing both the infectiousness of and the susceptibility to HIV infection [110]. Further studies are needed to unveil the cellular and molecular mechanisms by which CT and HIV interact with each other and their eukaryotic host during the early and later stages of the infection.

## 4. Co-Infection of *Chlamydia trachomatis* and Human Papillomavirus

### 4.1. Infection and Immunopathology of Human Papillomavirus

Papillomaviruses are non-enveloped, double-stranded DNA viruses from the family Papillomaviridae [119]. Low-risk HPV genotypes, such as HPV 6 and 11, are primarily associated with benign genital warts. These genotypes have low oncogenic potential [120]. On the other hand, high-risk HPV genotypes, particularly HPV 16 and 18, are strongly linked to the development of cervical, anogenital, and oropharyngeal cancers [120]. HPV is the most common STI in the world [1]. High-risk HPV infections are common globally, with a higher prevalence in sexually active individuals [120].

HPVs are transmitted primarily by direct physical contact and are capable of infecting the skin and mucosa [121]. The virus entry into undifferentiated epithelial cells depends on molecular interactions involving the viral particles and host receptors [120]. Structural studies on L1, the ~55 kDa major capsid protein of HPV, revealed that the presence of four heparan-sulfate proteoglycan-specific binding sites on the host eukaryotic cells is required for productive infection of papillomaviruses [120]. After endocytosis, the virus is transported within the small vesicles to the nucleus [120,122], and a series of interactions and structural changes of the vesicles allow decapsidation and the release of the viral genome near the nuclear membrane [120]. Finally, the viral genome enters the host cell nucleus to initiate replication [120,122]. For a productive life cycle, HPVs are dependent on squamous epithelial cell differentiation [122]. Papillomaviruses guarantee their multiplication and persistence by infecting undifferentiated basal cells [120,121,122].

HPV employs a different set of immune evasion mechanisms to that of HIV to overcome host immune responses and establish persistent infection [123,124,125]. Passively, HPV evades the immune system by its normal life cycle being located completely outside the epithelial basement membrane, not eliciting any danger signals [124]. Moreover, during early infection, HPV modulates viral gene expression to remain at a low-level, resulting in a minimal presentation of viral antigens [123,124,126]. This phenomenon is thought to favor immune tolerance rather than an effector T cell response that could effectively clear the disease [126]. Active immune evasion of HPV is mediated intracellularly by perturbing DNA methylation mechanisms to alter gene expression and disturbed protein functions, and extracellularly by interfering with immune cell networks from APCs to effector T cells [124,125]. Steinbach and Reimer [124] suggested that suppressed IFNγ and cGAS-STING responses inhibit the induction of an antiviral state. Later immune evasion mechanisms, such as downregulation of Toll-like receptors (TLRs), adhesion molecules on the infected host cell, and decreased chemokine production by the infected keratinocytes lead to reduced attraction of professional APCs and further cause a delay in triggering anti-HPV immune responses [123,124].

### 4.2. Epidemiology of Chlamydia trachomatis and Human Papillomavirus Co-Infections

Studies have reported an association between CT infection or history of CT infection with an increased risk of HPV infection in both men and women [37,46,127,128,129,130,131,132,133,134]. The risk factors for CT and HPV infections overlap, including becoming sexually active at a young age, an increased number of lifetime sexual partners both in females and their male partners, and non-condom contraception use [135,136,137,138,139]. Consequently, it is plausible to expect that people infected at some point with CT will have a higher prevalence of HPV compared to those without a history of CT infection. A study by Verteramo et al. [130] showed a significant correlation between the presence of HPV-DNA and concurrent genital infection with CT (*p* < 0.001) in cervical samples. Among HPV-positive patients co-infected with CT, a substantial proportion (67.56%) was infected with the high-risk HPV genotypes [130]. A study on Chinese women indicated a higher prevalence of high-risk HPV genotypes (22%) than low-risk HPV genotypes (2.5%) in those infected with CT [128]. Chronic, asymptomatic infection of the urogenital tract with CT could increase the odds of a persistent infection of HPV, which might be related to cervical hypertrophy and induction of squamous metaplasia, indicating a possible CT/HPV synergic effect on the etiology of cervical cancer [36]. Studies in Brazil and Mongolia reported that co-infection of HPV and CT may increase the risk for high-grade squamous intraepithelial lesions [47,140]. Another study by Escarcega-Tame et al. [48] suggested that CT/HPV co-infection is associated with cervical intraepithelial neoplasia and a higher risk of cervical cancer. Previous studies suggest that CT co-infection may be partly associated with infection of high-risk HPV genotypes [128,130,141], concurrent infection with multiple HPV types [37,128,140], and persistent infection of papillomaviruses [35,141].

### 4.3. Interaction of Chlamydia trachomatis with Human Papillomavirus in Co-Infections

**Figure 2:**
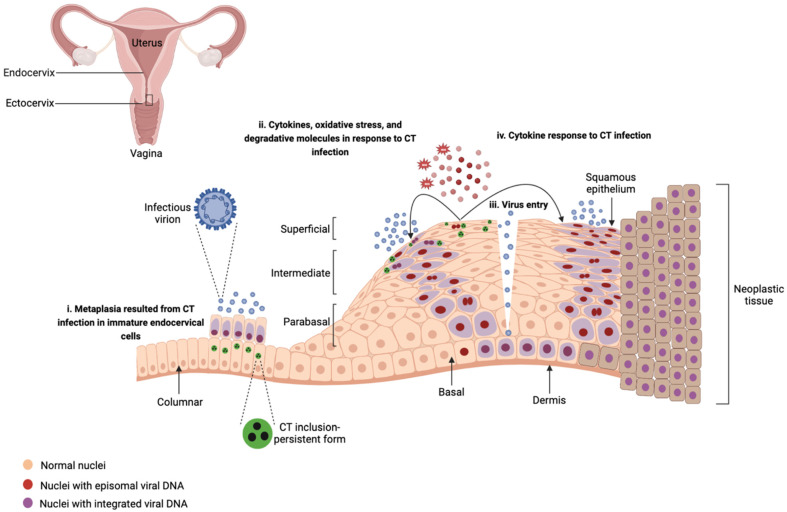
Interplay of *Chlamydia trachomatis* and host immune responses, and its impact on human papillomavirus infection in ectocervix and oncogenic transformation. *Chlamydia trachomatis* (CT) infection may increase susceptibility to human papillomavirus (HPV) infection through different strategies. (i) In the endocervix, infection of immature endocervical cells with CT leads to epithelial transformation (metaplasia). Metaplastic tissues are preferred by HPV for infection. (ii) In the ectocervix, CT infection in the squamous epithelium may lead to the release of reactive oxygen species (ROS), inflammatory cytokines, and molecules with degradative characteristics. ROS induces cellular DNA breaks that facilitate the integration of viral DNA. (iii) Chronic inflammation resulting from CT infection induces inflammatory responses that impair the epithelial integrity of the ectocervix and facilitates entry of HPV, therefore functioning as an entryway for the virions to the basal epithelium layer. (iv) Inflammatory cytokines released from CT-infected epithelial cells may influence HPV cell entry, replication, and enhance viral persistence. Created with BioRender.com.

Chronic inflammatory responses to CT infection may increase susceptibility to HPV infection. The inflammatory micro-environment caused by CT infection such as the release of ROS and molecules with degradative characteristics may impact HPV infection by (i) facilitating HPV cell entry, replication, and virus integration through enhancing cellular DNA breaks [37] and (ii) inducing damage to the epithelium and functioning as an entryway for the viral particles to the basal epithelium layer [35,36]. Lugo et al. [142] reported a predominance of pro-inflammatory cytokines in exfoliated cervical cells in CT-infected patients compared to healthy individuals emphasizing that the local inflammatory processes caused by CT may induce damage to the epithelium and clear the way for entry of HPV, viral persistence, and subsequent neoplastic progression. Moreover, CT has been shown to alter the characteristics of epithelial cells, which may result in increasing the viral load and facilitating the persistence of HPV [35,36,141,143]. CT can lead to epithelial transformation known as metaplasia by infecting immature endocervical cells [35]. Metaplastic epithelia are the preferred tissues by HPVs for infection [35]. CT infection may promote stemness and a preneoplastic molecular phenotype, and as a result, enhance the persistence of HPV by upregulating inflammatory responses such as IL-17 and NF-κB and mitogen-activated protein kinase (MAPK), and downregulating oxidative phosphorylation, RNA regulation, and RNA processing [18,50]. Figure 2 summarizes these findings, depicting how CT interacts with HPV during the course of a co-infection in ectocervix.

CT infection can disrupt the clearance of HPV infection by diminishing the immune response involved in the infection [143]. Indeed, chronic cervical inflammation related to CT infection inhibits cell-mediated immunity and its anti-apoptotic effect may influence the persistence and progression of the HPV infection [133,141,143]. CT may also stimulate HPV persistence by the functional decrease in APCs [36]. Lu et al. [50] reported that CT infection impairs the clearance of HPV by inducing downregulation of surface activation markers, impairment of the immune cytotoxicity of CD4+ and CD8+ T cells, and inhibition of cell-mediated immunity through modulation of the cervical immune response toward a T-helper 2 cell response.

CT/HPV co-infection and the potential synergistic role of these infections in cervical carcinogenesis were highlighted previously [35,46,47,129,140,144,145]. CT GroEL, a marker of CT persistence has been associated with an increased risk of cervical cancer [143]. CT GroEL may interfere with host apoptosis and senescence pathways, leading to the active proliferation of damaged cells infected by high-risk HPV types [143]. Moreover, higher expression of Matrix metalloproteinases (MMP)-9 and lower expression of RECK, and MMP-9/RECK imbalance were reported during high-risk HPV and CT infections, respectively [146,147]. MMPs are important enzymes in the tumor microenvironment and are associated with the progression of cervical intraepithelial neoplasia toward squamous cell carcinoma [146]. Lower expression of RECK, which has an inhibitory/regulatory effect on MMPs, is strongly associated with high-grade cervical lesions [146]. In an in vitro model of CT infection in ectocervical stem cells that were genetically manipulated to introduce E6E7 oncogenes and mimic HPV16 integration, HPV16 E6E7 interfered with CT development and induced persistence [18]. HPV E6 and E7 proteins degrade P53, a vital regulator of cell division and apoptosis, while also inhibiting the pRB protein, which controls cell-cycle progression, thereby facilitating the proliferation of the infected cell [148]. Remarkably, CT hindered HPV-induced mechanisms that maintain cellular and genome integrity, including mismatch repair (MMR) in the stem cells [18]. CT inhibited the DNA MMR pathway distinctly at the transcriptional and post-translational levels [18]. CT could disable MMR gene expression by proteasomal degradation of transcriptional factor E2F1 [18]. Previous studies indicated that CT causes centrosome amplification and multinucleation of the host cells [149,150] and that the effects of CT/HPV co-infection on the centrosome amplification were additive [150]. Multinucleation and centrosome amplification are considered hallmarks of cancer cells [150,151]. Knowlton et al. [152] reported that infection of mice with *Chlamydia muridarum* (CM) results in cellular proliferation, centrosome, and mitotic spindle defects promoting genomic instability. This supports the hypothesis that CT infection may prime the cervix for progression to neoplasia or exacerbate neoplastic lesions already present [152].

### 4.4. Conclusions

The evidence indicates that CT infection enhances the risk of HPV infection and persistence [46,50,147,153], and could be deleterious to cellular and genomic stability [18,50]. HPV and CT are both commonly asymptomatic and can evolve into a persistent infection which, added to co-infections, may be important co-factors for oncogenic transformation [154]. This highlights the importance of routine screening for HPV and CT infections in lowering the risk of cervical intraepithelial lesions and malignancies [36,128,130,133]. Our current knowledge is mainly limited to CT/HPV co-infection in the female genital tract, while a high prevalence of CT and HPV infections in anorectal and throat samples has been reported [12,154,155]. Therefore, further studies are needed to understand the contributing role of CT/HPV co-infection in anorectal and oropharyngeal malignancies.

## 5. Co-infections of *Chlamydia trachomatis* and Herpes Simplex Virus

### 5.1. Infection and Immunopathology of Herpes Simplex Virus

HSV is a double-stranded DNA virus from the family Herpesviridae that infects epithelial cells and neurons [145]. HSV-1 is transmitted by oral-to-oral contact and causes oral herpes, while HSV-2 is transmitted sexually and causes genital herpes. However, both viruses are capable of infecting and causing lesions at either of these sites [156,157,158]. Approximately, 3.7 billion individuals, which accounts for 67% of the global population under the age of 50 are believed to be infected with HSV-1. Additionally, an estimated 491 million people aged 15–49 (13%) worldwide have HSV-2 infection [158]. The importance of HSV as a sexually transmitted pathogen became evident as the risk of HIV acquisition was found to be at least three times higher in those with HSV-2 infection compared with un-infected individuals [159].

HSV transmission occurs through direct exposure of mucous membranes or abraded skin to the lesions or mucosal secretions of an individual with an active primary or recurrent infection [157]. The binding of HSV to host cell surface receptors and promoting entry into host cells requires viral glycoproteins (gB, gC, gD, gH, and gL), along with cellular receptors, such as nectin-1, nectin-2, herpes virus entry mediator (HVEM), or 3-O-sulfated HS (3-OS HS) [156,160]. Primary infection of HSV is characterized by viral replication at the site of inoculation, followed by retrograde axonal transport of the virus to corresponding sensory ganglia where infection may later reactivate or remain asymptomatic [157,161]. Recurrent HSV occurs in approximately 60% of those infected with HSV-1 and 90% with HSV-2 [159,160].

HSV has evolved several strategies to evade the innate and adaptive immune responses. Strategies employed to modulate the innate immune responses are directed at several antiviral host defenses, particularly cytokines such as IFN-α/β, the complement system, NK cell function, DC function, and antigen-dependent responses [162,163]. Strategies to modulate the adaptive immune responses are directed at humoral immune responses and T-cell responses [162]. These are crucial for effective virus propagation and establishing an environment for virus replication and latency [162,163]. White et al. [164] hypothesized that herpesvirus latency may impact resistance to secondary infections and the outcome of the secondary infections in humans. The explanation for this hypothesis relies on the premise of a mutually beneficial symbiotic relationship with the host that fosters protection against secondary infections. The acquisition of multiple herpesviruses during early life is associated with increased resistance to secondary infections, a phenomenon that is thought to be mediated by latency-driven immune responses [164].

### 5.2. Epidemiology of Chlamydia trachomatis and Herpes Simplex Virus Co-Infections

The majority of reports on CT/HSV co-infections are from those with urogenital sequelae; however, there are rare reports of the co-infection in oral and ocular samples [45,165,166,167,168,169,170]. Most of the epidemiological studies on CT/HSV co-infection rely on serological data [171], making it difficult to determine whether or not both pathogens were present simultaneously in the genital tract at any given time. Several studies described a high prevalence of CT and HSV seropositivity among men and women [172,173,174,175]. In a group of 35 patients with cervical dysplasia or cervical carcinoma, IgA antibodies to HSV were detected in the cervical secretions of 10 (28%), from which nine had IgA antibodies to CT [172]. An examination of 88 students from tertiary institutions revealed that 9.1% of the participants had IgG antibodies present for both CT and HSV [175]. Vetter et al. [170] suggested that seropositive women for CT and HSV-2 may experience more severe outcomes, such as endometritis and salpingitis, than those infected with CT or HSV-2 alone. A higher incidence of spontaneous abortions was found in women co-infected with CT and HSV-2 than those infected with CT or HSV-2 alone [176].

### 5.3. Interaction of Chlamydia trachomatis with Herpes Simplex Virus in Co-Infections

There are diverse observations of the impact of CT on HSV progeny during co-infection. An in vitro model of CT/HSV-2 co-infection in HeLa cells suggested that pre-infection of HeLa cells with CT increases HSV-2 replication by up to 40% [177]. In contrast, Deka et al. [19] suggested that CT co-infection in HeLa cells does not significantly affect HSV-2 replication. In an in vivo study, female BALB/c mice vaginally pre-infected with CM, three or nine days prior to HSV-2 infection, conferred significant protection from HSV-2-induced neurologic disease and significantly reduced viral recovery compared with control animals infected only with HSV-2 [171]. This super-infection scenario benefited the host when mice were pre-infected with CM either prior to, simultaneously with, or shortly after the challenge with HSV-2 [171]. The protective effect was lost when chlamydial shedding from the genital tract ceased, either naturally or due to antibiotic treatment [171]. Interestingly, replacing live CM with UV-irradiated, replication-incompetent CM, failed to elicit protection from HSV-2-induced mortality [171]. These observations could be partly explained by the role of CM in eliciting protection from subsequent HSV-2 challenge through activation of TLR-2, enhancing IFN-β secretion, and CPAF-dependent degradation of nectin-1—the primary co-receptor for HSV-2 in the genital tract [171]. It has been shown that TLR2 plays an important role in controlling HSV-2 infection and IFN-β is a strong inhibitor of HSV-2 infection [178,179,180].

Interestingly, co-infection of CT and HSV leads CT into a viable but non-cultivable state, which is the hallmark of persistence [19,181]. Previously, it has been shown that CT/HSV co-infection enhances the accumulation of CT GroEL [19], which is a marker of chlamydial persistence. A study using HeLa and HEC-1B cells revealed that during CT genovar E and HSV-1/2 co-infection, the attachment and/or entry of the viral particles to the host cells is enough to transmit a signal triggering CT persistence [51]. Notably, using cycloheximide, an inhibitor of eukaryotic protein synthesis in culture or UV-inactivated HSV led to CT persistence, suggesting that productive viral replication is not required for the induction of chlamydial persistence and that early events during HSV-2 infection are sufficient for CT persistence [51]. Exposure of CT-infected cells to a soluble recombinant HSV-2 gD: Fc fusion protein decreased the production of infectious EBs to a degree similar to that observed in co-infected cultures with HSV, which indicates that contact between the HSV-2 gD protein and the host cell surface is sufficient to induce chlamydial persistence [181]. Musarrat et al. [182] and Hall et al. [183] reported that the interaction of HSV gD with the host cell receptor, nectin-1, triggers the release of intracellular calcium stores at the plasma membrane [182,183]. The interplay between calcium and ROS signaling pathways leads to changes in ROS accumulation in the eukaryotic cells [183]. Therefore, HSV attachment to the host cell may induce a transient oxidative stress condition that ultimately causes chlamydial persistence [183]. A study by Prusty et al. [52] on CT genovar L2 and human herpesvirus-6 (HHV-6) co-infected cells suggested that HHV6 infection modulates cellular glutathione reductase (GSR) activity leading to increased oxidative stress and decreased levels of reduced glutathione (GSH). A decrease in the level of GSH and concomitant elevation of nicotinamide adenine dinucleotide phosphate (NADPH) levels in cells only infected with the virus suggest that HHV6 infection interferes with GSR activity and thus causes an imbalance in the detoxification of ROS, resulting in CT persistence [52]. Similar findings were observed for CT/HSV1 co-infection [52].

### 5.4. Conclusions

Given that both CT and HSV can cause long-term asymptomatic infections and influence the immune responses of the host [19], we assume a high prevalence of CT/HSV co-infection in sexually active individuals. However, the current understanding of the epidemiology relies heavily on serological data, necessitating further investigations utilizing molecular diagnostic techniques to elucidate the occurrence of CT/HSV co-infection in diverse populations. The difficulty with serology is that (i) the serological test does not directly measure the disease itself or its underlying cause but rather assesses the patient’s immune system [184], and (ii) some immunocompromised patients will not mount an adequate antibody response to infection [185]. There is conflicting evidence regarding the impact of CT on HSV infection in laboratory settings. Nevertheless, the only in vivo study currently available speculates that the (i) activation of TLR-2 and secretion of IFN-β result from an early CM infection, and (ii) the chlamydial protease, CPAF, can degrade the HSV co-receptor on the host cell, playing roles in reduced viral recovery. The mechanism by which HSV causes CT persistence can be explained based on the interaction of HSV gD protein with the nectin-1 receptor on the host cells leading to an impaired ROS detoxification mechanism. Further studies using transcriptomics could help to achieve a better profile of the interplay between CT, HSV, and their eukaryotic host cells.

## 6. Concluding Remarks

CT infection of urogenital and rectal epithelial cells can facilitate viral infection through multiple mechanisms, including (i) damaging the mucus membrane and decreasing epithelial integrity that opens a route for infection, (ii) direct interaction with the viruses’ host cells, and (iii) modulating the host immune responses. Future studies, including using techniques such as transcriptomics, could lead to a better understanding of the interaction of the pathogens with each other and their eukaryotic host during co-infections. Furthermore, there is a pressing need for in vitro and in vivo studies to elucidate the role of CT co-infection in the acquisition, pathogenesis, viral load, transmission, disease progression, and severity of sexually transmitted viral infections. Such investigations will contribute to a clearer understanding of the clinical implications of CT co-infections with viral STIs.

At the epidemiological level, we highlight the substantial gaps in our knowledge of the prevalence of co-infections of CT with viral STIs among different populations and factors associated with the prevalence of these co-infections. It is crucial to conduct further studies employing molecular techniques to generate robust epidemiological evidence that can enhance our understanding of the clinical significance of these co-infections.

Overall, advancing our understanding of the interactions between CT and viral STIs in co-infections is vital for informing preventive strategies, developing targeted interventions, and improving clinical management in order to mitigate the burden of these infections on global public health.

## Data Availability

Data sharing not applicable.

## References

[1] Van Gerwen O.T., Muzny C.A., Marrazzo J.M. (2022). Sexually transmitted infections and female reproductive health. Nat. Microbiol..

[2] Unemo M., Lahra M.M., Escher M., Eremin S., Cole M.J., Galarza P., Ndowa F., Martin I., Dillon J.-A.R., Galas M. (2021). WHO global antimicrobial resistance surveillance for Neisseria gonorrhoeae 2017–18: A retrospective observational study. Lancet Microbe.

[3] (CDC) Centers for Disease Control and Prevention (2022). Incidence, Prevalence, and Cost of Sexually Transmitted Infections in the United States.

[4] Choudhry S., Ramachandran V., Das S., Bhattacharya S., Mogha N.S. (2010). Characterization of patients with multiple sexually transmitted infections: A hospital-based survey. Indian J. Sex. Transm. Dis. AIDS.

[5] Poudel K.C., Poudel-Tandukar K., Palmer P.H., Mizoue T., Jimba M., Kobayashi J., Acharya B., Pandey B.D., Oka S. (2015). Coinfection of Sexually Transmitted Infections among HIV-Positive Individuals: Cross-Sectional Results of a Community-Based Positive Living with HIV (POLH) Study in Nepal. J. Int. Assoc. Provid. AIDS Care (JIAPAC).

[6] Pakianathan M.R., Ross J.D.C., McMillan A. (1996). Characterizing patients with multiple sexually acquired infections: A multivariate analysis. Int. J. STD AIDS.

[7] E Broad C., Furegato M., A Harrison M., Pond M.J., Tan N., Okala S., Fuller S.S., Harding-Esch E.M., Sadiq S.T. (2020). High prevalence of coinfection of azithromycin-resistant *Mycoplasma genitalium* with other STIs: A prospective observational study of London-based symptomatic and STI-contact clinic attendees. Sex. Transm. Infect..

[8] Lee S.J., Jang T.S., Jeon J., Kim J.K. (2022). Coinfections with multiple sexually transmitted pathogens in Republic of Korea, 2018. J. Clin. Lab. Anal..

[9] Kalichman S.C., Pellowski J., Turner C. (2011). Prevalence of sexually transmitted co-infections in people living with HIV/AIDS: Systematic review with implications for using HIV treatments for prevention. Sex. Transm. Infect..

[10] van Veen M.G., Koedijk F.D.H., van der Sande M.A.B. (2010). STD Coinfections in The Netherlands: Specific Sexual Networks at Highest Risk. Sex. Transm. Dis..

[11] de Coul E.O., Warning T., Koedijk F.D.H., Dutch STI clinics (2013). Sexual behaviour and sexually transmitted infections in sexually transmitted infection clinic attendees in the Netherlands, 2007–2011. Int. J. STD AIDS.

[12] Ye Z.-H., Chen S., Liu F., Cui S.-T., Liu Z.-Z., Jiang Y.-J., Hu Q.-H. (2022). Patterns of Sexually Transmitted Co-infections and Associated Factors among Men Who Have Sex With Men: A Cross-Sectional Study in Shenyang, China. Front. Public Health.

[13] Góes S.d.S., Fonseca R.R.d.S., Avelino M.E.d.S., Lima S.S., de Lima M.S.G.A., Laurentino R.V., Queiroz M.A.F., Freitas F.B., Vallinoto A.C.R., Ishak R. (2022). Exposure to *Chlamydia trachomatis* Infection in Individuals Who Are Newly Diagnosed with HIV and Antiretroviral-Naïve from Belém, Northern Brazil. Vaccines.

[14] Sentís A., Martin-Sanchez M., Arando M., Vall M., Barbera M.J., Ocaña I., Cordón A.G., Alsina M., Martin-Ezquerra G., Knobel H. (2019). Sexually transmitted infections in young people and factors associated with HIV coinfection: An observational study in a large city. BMJ Open.

[15] Ginindza T.G., Stefan C.D., Tsoka-Gwegweni J.M., Dlamini X., Jolly P.E., Weiderpass E., Broutet N., Sartorius B. (2017). Prevalence and risk factors associated with sexually transmitted infections (STIs) among women of reproductive age in Swaziland. Infect. Agents Cancer.

[16] Abbai N.S., Wand H., Ramjee G. (2013). Sexually Transmitted Infections in Women Participating in a Biomedical Intervention Trial in Durban: Prevalence, Coinfections, and Risk Factors. J. Sex. Transm. Dis..

[17] Nguyen P.T.T., Pham H.V., Van D.H., Van Pham L., Nguyen H.T., Van Nguyen H. (2022). Randomized controlled trial of the relative efficacy of high-dose intravenous ceftriaxone and oral cefixime combined with doxycycline for the treatment of Chlamydia trachomatis and Neisseria gonorrhoeae co-infection. BMC Infect. Dis..

[18] Koster S., Gurumurthy R.K., Kumar N., Prakash P.G., Dhanraj J., Bayer S., Berger H., Kurian S.M., Drabkina M., Mollenkopf H.-J. (2022). Modelling Chlamydia and HPV co-infection in patient-derived ectocervix organoids reveals distinct cellular reprogramming. Nat. Commun..

[19] Deka S., Vanover J., Dessus-Babus S., Whittimore J., Howett M.K., Wyrick P.B., Schoborg R.V. (2006). Chlamydia trachomatis enters a viable but non-cultivable (persistent) state within herpes simplex virus type 2 (HSV-2) co-infected host cells. Cell. Microbiol..

[20] Cornelisse V.J., Sherman C.J., Hocking J.S., Williams H., Zhang L., Chen M.Y., Bradshaw C.S., Bellhouse C., Fairley C.K., Chow E.P.F. (2017). Concordance of chlamydia infections of the rectum and urethra in same-sex male partnerships: A cross-sectional analysis. BMC Infect. Dis..

[21] Cina M., Baumann L., Egli-Gany D., Halbeisen F.S., Ali H., Scott P., Low N. (2019). *Mycoplasma genitalium* incidence, persistence, concordance between partners and progression: Systematic review and meta-analysis. Sex. Transm. Infect..

[22] Khan A., Fortenberry J.D., Juliar B.E., Tu W., Orr D.P., Batteiger B.E. (2005). The Prevalence of Chlamydia, Gonorrhea, and Trichomonas in Sexual Partnerships: Implications for Partner Notification and Treatment. Sex. Transm. Dis..

[23] Schillinger J.A.M., Katz B.P., Markowitz L.E., Braslins P.G., Shrier L.A., Madico G., Van Der Pol B., Orr D.P., Rice P.A., Batteiger B.E. (2016). Genotype-Specific Concordance of Chlamydia trachomatis Genital Infection Within Heterosexual Partnerships. Sex. Transm. Dis..

[24] Van Der Pol B. (2007). *Trichomonas vaginalis* Infection: The Most Prevalent Nonviral Sexually Transmitted Infection Receives the Least Public Health Attention. Clin. Infect. Dis..

[25] O’connell C.M., Ferone M.E. (2016). Chlamydia trachomatis Genital Infections. Microb. Cell.

[26] Rodrigues R., Sousa C., Vale N. (2022). *Chlamydia trachomatis* as a Current Health Problem: Challenges and Opportunities. Diagnostics.

[27] (WHO) World Health Organization (2022). Sexually Transmitted Infections (STIs).

[28] Rowley J., Vander Hoorn S., Korenromp E., Low N., Unemo M., Abu-Raddad L.J., Chico R.M., Smolak A., Newman L., Gottlieb S. (2019). Chlamydia, gonorrhoea, trichomoniasis and syphilis: Global prevalence and incidence estimates. Bull. World Health Organ..

[29] Bastidas R.J., Elwell C.A., Engel J.N., Valdivia R.H. (2013). Chlamydial Intracellular Survival Strategies. Cold Spring Harb. Perspect. Med..

[30] Wyrick P.B. (2010). *Chlamydia trachomatis* Persistence In Vitro: An Overview. J. Infect. Dis..

[31] Rajić J., Inic-Kanada A., Stein E., Dinić S., Schuerer N., Uskoković A., Ghasemian E., Mihailović M., Vidaković M., Grdović N. (2017). Chlamydia trachomatis Infection Is Associated with E-Cadherin Promoter Methylation, Downregulation of E-Cadherin Expression, and Increased Expression of Fibronectin and α-SMA—Implications for Epithelial-Mesenchymal Transition. Front. Cell. Infect. Microbiol..

[32] Ghasemian E., Inic-Kanada A., Collingro A., Tagini F., Stein E., Alchalabi H., Schuerer N., Keše D., Babiker B.E., Borel N. (2018). Detection of Chlamydiaceae and Chlamydia-like organisms on the ocular surface of children and adults from a trachoma-endemic region. Sci. Rep..

[33] Geisler W.M. (2010). Duration of Untreated, Uncomplicated *Chlamydia trachomatis* Genital Infection and Factors Associated with Chlamydia Resolution: A Review of Human Studies. J. Infect. Dis..

[34] Champredon D., E Bellan S., Delva W., Hunt S., Shi C.-F., Smieja M., Dushoff J. (2015). The effect of sexually transmitted co-infections on HIV viral load amongst individuals on antiretroviral therapy: A systematic review and meta-analysis. BMC Infect. Dis..

[35] Wohlmeister D., Vianna D.R.B., Helfer V.E., Gimenes F., Consolaro M.E.L., Barcellos R.B., Rossetti M.L., Calil L.N., Buffon A., Pilger D.A. (2016). Association of human papillomavirus and Chlamydia trachomatis with intraepithelial alterations in cervix samples. Memórias Do Inst. Oswaldo Cruz.

[36] Silva J., Cerqueira F., Medeiros R. (2014). Chlamydia trachomatis infection: Implications for HPV status and cervical cancer. Arch. Gynecol. Obstet..

[37] Seraceni S., Campisciano G., Contini C., Comar M. (2016). HPV genotypes distribution in Chlamydia trachomatis co-infection in a large cohort of women from north-east Italy. J. Med. Microbiol..

[38] Schust D.J., Ibana J.A., Buckner L.R., Ficarra M., Sugimoto J., Amedee A.M., Quayle A.J. (2012). Potential mechanisms for increased HIV-1 transmission across the endocervical epithelium during C. trachomatis infection. Curr. HIV Res..

[39] Buckner L.R., Amedee A.M., Albritton H.L., Kozlowski P.A., Lacour N., McGowin C.L., Schust D.J., Quayle A.J. (2016). Chlamydia trachomatis Infection of Endocervical Epithelial Cells Enhances Early HIV Transmission Events. PLoS ONE.

[40] Sun J., Kintner J., Schoborg R.V. (2008). The host adherens junction molecule nectin-1 is downregulated in Chlamydia trachomatis-infected genital epithelial cells. Microbiology.

[41] Prozialeck W.C., Fay M.J., Lamar P.C., Pearson C.A., Sigar I., Ramsey K.H. (2002). *Chlamydia trachomatis* Disrupts N-Cadherin-Dependent Cell-Cell Junctions and Sequesters β-Catenin in Human Cervical Epithelial Cells. Infect. Immun..

[42] Chkhartishvili N., Dvali N., Khechiashvili G., Sharvadze L., Tsertsvadze T. (2010). High seroprevalence of Chlamydia trachomatis in newly diagnosed human immunodeficiency virus patients in georgia. Georgian Med. News.

[43] Mafokwane T.M., Samie A. (2016). Prevalence of chlamydia among HIV positive and HIV negative patients in the Vhembe District as detected by real time PCR from urine samples Microbiology. BMC Res. Notes.

[44] Bhattar S., Bhalla P., Chadha S., Tripathi R., Kaur R., Sardana K. (2013). *Chlamydia trachomatis* Infection in HIV-Infected Women: Need for Screening by a Sensitive and Specific Test. Infect. Dis. Obstet. Gynecol..

[45] Fageeh W.M.K. (2013). Sexually transmitted infections among patients with herpes simplex virus at King Abdulaziz University Hospital. BMC Res. Notes.

[46] Ssedyabane F., Amnia D.A., Mayanja R., Omonigho A., Ssuuna C., Najjuma J.N., Freddie B. (2019). HPV-Chlamydial Coinfection, Prevalence, and Association with Cervical Intraepithelial Lesions: A Pilot Study at Mbarara Regional Referral Hospital. J. Cancer Epidemiol..

[47] de Abreu A.L.P., Teixeira J.J.V., Nogara P.R.B., da Silva M.C., Tognim M.C.B., Zanko R.L., Souza R.P., Consolaro M.E.L., Uchimura N.S., Gimenes F. (2012). Short Report: Molecular Detection of HPV and Chlamydia trachomatis Infections in Brazilian Women with Abnormal Cervical Cytology. Am. J. Trop. Med. Hyg..

[48] Escarcega-Tame M.A., López-Hurtado M., Escobedo-Guerra M.R., Reyes-Maldonado E., Castro-Escarpulli G., Guerra-Infante F.M. (2020). Co-infection between genotypes of the human papillomavirus and Chlamydia trachomatis in Mexican women. Int. J. STD AIDS.

[49] Henning T.R., Butler K., Hanson D., Sturdevant G., Ellis S., Sweeney E.M., Mitchell J., Deyounks F., Phillips C., Farshy C. (2014). Increased Susceptibility to Vaginal Simian/Human Immunodeficiency Virus Transmission in Pig-tailed Macaques Coinfected with Chlamydia trachomatis and Trichomonas vaginalis. J. Infect. Dis..

[50] Lu Y., Wu Q., Central S.Z., Li H., Shanghai W., Central Z., Lingting H., Zhabei J.S. Chlamydia Trachomatis Enhances HPV Persistence through Immune Modulation. Res. Square.

[51] Deka S., Vanover J., Sun J., Kintner J., Whittimore J., Schoborg R.V. (2007). An early event in the herpes simplex virus type-2 replication cycle is sufficient to induce Chlamydia trachomatis persistence. Cell. Microbiol..

[52] Prusty B.K., Böhme L., Bergmann B., Siegl C., Krause E., Mehlitz A., Rudel T. (2012). Imbalanced Oxidative Stress Causes Chlamydial Persistence during Non-Productive Human Herpes Virus Co-Infection. PLoS ONE.

[53] Prusty B.K., Siegl C., Hauck P., Hain J., Korhonen S.J., Hiltunen-Back E., Puolakkainen M., Rudel T. (2013). Chlamydia trachomatis Infection Induces Replication of Latent HHV-6. PLoS ONE.

[54] Di Pietro M., Filardo S., Simonelli I., Pasqualetti P., Sessa R. (2022). Cervicovaginal Microbiota Composition in *Chlamydia trachomatis* Infection: A Systematic Review and Meta-Analysis. Int. J. Mol. Sci..

[55] Di Pietro M., Filardo S., Grazia Porpora M., Recine N., Agnese Latino M., Sessa R. (2018). HPV/Chlamydia trachomatis co-infection: Metagenomic analysis of cervical microbiota in asymptomatic women. New Microbiol..

[56] Tamarelle J., de Barbeyrac B., Le Hen I., Thiébaut A., Bébéar C., Ravel J., Delarocque-Astagneau E. (2018). Vaginal microbiota composition and association with prevalent *Chlamydia trachomatis* infection: A cross-sectional study of young women attending a STI clinic in France. Sex. Transm. Infect..

[57] Lewis F.M.T., Bernstein K.T.P., Aral S.O. (2017). Vaginal Microbiome and Its Relationship to Behavior, Sexual Health, and Sexually Transmitted Diseases. Obstet. Gynecol..

[58] Nijhuis R.H.T., Duinsbergen R.G., Pol A., Godschalk P.C.R. (2021). Prevalence of Chlamydia trachomatis, Neisseria gonorrhoeae, Mycoplasma genitalium and Trichomonas vaginalis including relevant resistance-associated mutations in a single center in the Netherlands. Eur. J. Clin. Microbiol. Infect. Dis..

[59] Perry M.D., Jones S., Bertram A., de Salazar A., Barrientos-Durán A., Schiettekatte G., Lewinski M., Arcenas R., Hansra A., Njoya M. (2023). The prevalence of Mycoplasma genitalium (MG) and Trichomonas vaginalis (TV) at testing centers in Belgium, Germany, Spain, and the UK using the cobas TV/MG molecular assay. Eur. J. Clin. Microbiol. Infect. Dis..

[60] Jongen V.W., van der Loeff M.F.S., Botha M.H., Sudenga S.L., Abrahamsen M.E., Giuliano A.R. (2021). Incidence and risk factors of C. trachomatis and N. gonorrhoeae among young women from the Western Cape, South Africa: The EVRI study. PLoS ONE.

[61] Seo Y., Choi K.-H., Lee G. (2021). Characterization and Trend of Co-Infection with *Neisseria gonorrhoeae* and *Chlamydia trachomatis* from the Korean National Infectious Diseases Surveillance Database. World J. Men’s Health.

[62] Solomon A.W., Burton M.J., Gower E.W., Harding-Esch E.M., Oldenburg C.E., Taylor H.R., Traoré L. (2022). Trachoma. Nat. Rev. Dis. Prim..

[63] Heijer C.D.J.D., A Hoebe C.J.P., Driessen J.H.M., Wolffs P., Broek I.V.F.v.D., Hoenderboom B.M., Williams R., de Vries F., Dukers-Muijrers N.H.T.M. (2019). Chlamydia trachomatis and the Risk of Pelvic Inflammatory Disease, Ectopic Pregnancy, and Female Infertility: A Retrospective Cohort Study among Primary Care Patients. Clin. Infect. Dis..

[64] de Vries H.J. (2019). Lymphoganuloma venereum in the Western world, 15 years after its re-emergence. Curr. Opin. Infect. Dis..

[65] Witkin S.S., Minis E., Athanasiou A., Leizer J., Linhares I.M. (2017). Chlamydia trachomatis: The Persistent Pathogen. Clin. Vaccine Immunol..

[66] Elwell C., Mirrashidi K., Engel J. (2016). Chlamydia cell biology and pathogenesis. Nat. Rev. Microbiol..

[67] Carabeo R.A., Grieshaber S.S., Fischer E., Hackstadt T. (2002). *Chlamydia trachomatis* Induces Remodeling of the Actin Cytoskeleton during Attachment and Entry into HeLa Cells. Infect. Immun..

[68] Moore E.R., Ouellette S.P. (2014). Reconceptualizing the chlamydial inclusion as a pathogen-specified parasitic organelle: An expanded role for Inc proteins. Front. Cell. Infect. Microbiol..

[69] AbdelRahman Y.M., Belland R.J. (2005). The chlamydial developmental cycle. FEMS Microbiol. Rev..

[70] Murray S.M., McKay P.F. (2021). Chlamydia trachomatis: Cell biology, immunology and vaccination. Vaccine.

[71] Mpiga P., Ravaoarinoro M. (2006). Chlamydia trachomatis persistence: An update. Microbiol. Res..

[72] Gérard H.C., A Whittum-Hudson J., Schumacher H., Hudson A.P. (2004). Differential expression of three Chlamydia trachomatis hsp60-encoding genes in active vs. persistent infections. Microb. Pathog..

[73] Illingworth M., Hooppaw A.J., Ruan L., Fisher D.J., Chen L. (2017). Biochemical and Genetic Analysis of the Chlamydia GroEL Chaperonins. J. Bacteriol..

[74] Brunham R.C., Rey-Ladino J. (2005). Immunology of Chlamydia infection: Implications for a Chlamydia trachomatis vaccine. Nat. Rev. Immunol..

[75] Redgrove K.A., McLaughlin E.A. (2014). The Role of the Immune Response in Chlamydia trachomatis Infection of the Male Genital Tract: A Double-Edged Sword. Front. Immunol..

[76] Chen A.L., Johnson K.A., Lee J.K., Sütterlin C., Tan M. (2012). CPAF: A Chlamydial Protease in Search of an Authentic Substrate. PLoS Pathog..

[77] Prusty B.K., Chowdhury S.R., Gulve N., Rudel T. (2018). Peptidase Inhibitor 15 (PI15) Regulates Chlamydial CPAF Activity. Front. Cell. Infect. Microbiol..

[78] Tan M., Sütterlin C. (2014). The *Chlamydia* protease CPAF: Caution, Precautions and Function. Pathog. Dis..

[79] Snavely E.A., Kokes M., Dunn J.D., Saka H.A., Nguyen B.D., Bastidas R.J., McCafferty D.G., Valdivia R.H. (2014). Reassessing the role of the secreted protease CPAF in *Chlamydia trachomatis* infection through genetic approaches. Pathog. Dis..

[80] Patton M.J., McCorrister S., Grant C., Westmacott G., Fariss R., Hu P., Zhao K., Blake M., Whitmire B., Yang C. (2016). Chlamydial Protease-Like Activity Factor and Type III Secreted Effectors Cooperate in Inhibition of p65 Nuclear Translocation. mBio.

[81] Schott B.H., Antonia A.L., Wang L., Pittman K.J., Sixt B.S., Barnes A.B., Valdivia R.H., Ko D.C. (2020). Modeling of variables in cellular infection reveals CXCL10 levels are regulated by human genetic variation and the Chlamydia-encoded CPAF protease. Sci. Rep..

[82] Rodel J., Groh A., Vogelsang H., Lehmann M., Hartmann M., Straube E. (1998). Beta Interferon Is Produced by *Chlamydia trachomatis* -Infected Fibroblast-Like Synoviocytes and Inhibits Gamma Interferon-Induced HLA-DR Expression. Infect. Immun..

[83] Wong W.F., Chambers J.P., Gupta R., Arulanandam B.P. (2019). *Chlamydia* and Its Many Ways of Escaping the Host Immune System. J. Pathog..

[84] Rajeeve K., Das S., Prusty B.K., Rudel T. (2018). Chlamydia trachomatis paralyses neutrophils to evade the host innate immune response. Nat. Microbiol..

[85] Deeks S.G., Overbaugh J., Phillips A., Buchbinder S. (2015). HIV infection. Nat. Rev. Dis. Prim..

[86] McLaren P.J., Fellay J. (2021). HIV-1 and human genetic variation. Nat. Rev. Genet..

[87] (WHO) World Health Organization (2023). HIV Data and Statistics.

[88] Taylor B.S., Sobieszczyk M.E., McCutchan F.E., Hammer S.M. (2008). The Challenge of HIV-1 Subtype Diversity. N. Engl. J. Med..

[89] Shi Y., Su J., Chen R., Wei W., Yuan Z., Chen X., Wang X., Liang H., Ye L., Jiang J. (2022). The Role of Innate Immunity in Natural Elite Controllers of HIV-1 Infection. Front. Immunol..

[90] Yasen A., Herrera R., Rosbe K., Lien K., Tugizov S.M. (2018). HIV internalization into oral and genital epithelial cells by endocytosis and macropinocytosis leads to viral sequestration in the vesicles. Virology.

[91] Chinen J., Shearer W.T. (2002). Molecular virology and immunology of HIV infection. J. Allergy Clin. Immunol..

[92] Schiff A.E., Linder A.H., Luhembo S.N., Banning S., Deymier M.J., Diefenbach T.J., Dickey A.K., Tsibris A.M., Balazs A.B., Cho J.L. (2021). T cell-tropic HIV efficiently infects alveolar macrophages through contact with infected CD4+ T cells. Sci. Rep..

[93] Mohamed H., Gurrola T., Berman R., Collins M., Sariyer I.K., Nonnemacher M.R., Wigdahl B. (2022). Targeting CCR5 as a Component of an HIV-1 Therapeutic Strategy. Front. Immunol..

[94] Wilen C.B., Tilton J.C., Doms R.W. (2012). HIV: Cell Binding and Entry. Cold Spring Harb. Perspect. Med..

[95] Más V., Melero J.A. (2013). Entry of Enveloped Viruses into Host Cells: Membrane Fusion. Subcell Biochem..

[96] Dhawan B., Vajpayee M., Ghosh A., Chaudhry R., Sreenivas V. (2011). Genital mycoplasma & Chlamydia trachomatis infections in treatment naïve HIV-1 infected adults. Indian J. Med. Res..

[97] Cameron D., D’Costa L., Maitha G., Cheang M., Piot P., Simonsen J., Ronald A., Gakinya M., Ndinya-Achola J., Brunham R. (1989). Female to male transmission of human immunodeficiency virus type 1: Risk factors for seroconversion in men. Lancet.

[98] Johnson L.F., Lewis D.A. (2008). The Effect of Genital Tract Infections on HIV-1 Shedding in the Genital Tract: A Systematic Review and Meta-Analysis. Sex. Transm. Dis..

[99] Sherwal B., Nayyar C., Chander R., Gupta P. (2014). Co-infection of human immunodeficiency virus and sexually transmitted infections in circumcised and uncircumcised cases in India. Indian J. Sex. Transm. Dis. AIDS.

[100] Grosskurth H., Todd J., Mwijarubi E., Mayaud P., Nicoll A., Ka-Gina G., Newell J., Mabey D., Hayes R., Mosha F. (1995). Impact of improved treatment of sexually transmitted diseases on HIV infection in rural Tanzania: Randomised controlled trial. Lancet.

[101] Rotchford K., Strum W.A., Wilkinson D. (2000). Effect of Coinfection With STDs and of STD Treatment on HIV Shedding in Genital-Tract Secretions: Systematic Review and Data Synthesis. Sex. Transm. Dis..

[102] Mayer K.H., Venkatesh K.K. (2011). Interactions of HIV, Other Sexually Transmitted Diseases, and Genital Tract Inflammation Facilitating Local Pathogen Transmission and Acquisition. Am. J. Reprod. Immunol..

[103] Ghys P.D., Fransen K., Diallo M.O., Ettiègne-Traoré V., Coulibaly I.-M., Yeboué K.M., Kalish M.L., Maurice C., Whitaker J.P., Greenberg A.E. (1997). The associations between cervicovaginal HIV shedding, sexually transmitted diseases and immunosuppression in female sex workers in Abidjan, Côte d’Ivoire. AIDS.

[104] Katz D.A., Dombrowski J.C., Bell T.R., Kerani R.P., Golden M.R. (2016). HIV Incidence Among Men Who Have Sex with Men after Diagnosis with Sexually Transmitted Infections. Sex. Transm. Dis..

[105] Bernstein K.T.P., Marcus J.L., Nieri G.B., Philip S.S., Klausner J.D. (2010). Rectal Gonorrhea and Chlamydia Reinfection Is Associated with Increased Risk of HIV Seroconversion. Am. J. Ther..

[106] Naresh A., Beigi R., Woc-Colburn L., Salata R.A. (2009). The Bidirectional Interactions of Human Immunodeficiency Virus-1 and Sexually Transmitted Infections. Infect. Dis. Clin. Pract..

[107] Pathela P., Braunstein S.L., Blank S., Schillinger J.A. (2013). HIV Incidence among Men with and Those without Sexually Transmitted Rectal Infections: Estimates from Matching against an HIV Case Registry. Clin. Infect. Dis..

[108] McClelland R.S., Wang C.C., Mandaliya K., Overbaugh J., Reiner M.T., Panteleeff D.D., Lavreys L., Ndinya-Achola J., Bwayo J.J., Kreiss J.K. (2001). Treatment of cervicitis is associated with decreased cervical shedding of HIV-1. AIDS.

[109] Sadiq S.T., Taylor S., Copas A.J., Bennett J., Kaye S., Drake S.M., Kirk S., Pillay D., Weller I.V.D. (2005). The effects of urethritis on seminal plasma HIV-1 RNA loads in homosexual men not receiving antiretroviral therapy. Sex. Transm. Infect..

[110] Galvin S.R., Cohen M.S. (2004). The role of sexually transmitted diseases in HIV transmission. Nat. Rev. Microbiol..

[111] A Røttingen J., Cameron D.W., Garnett G.P. (2001). A Systematic Review of the Epidemiologic Interactions between Classic Sexually Transmitted Diseases and HIV. Sex. Transm. Dis..

[112] Cohen C.R., Plummer F.A., Mugo N., Maclean I., Shen C., Bukusi E.A., Irungu E., Sinei S., Bwayo J., Brunham R.C. (1999). Increased interleukin-10 in the endocervical secretions of women with non-ulcerative sexually transmitted diseases: A mechanism for enhanced HIV-1 transmission?. AIDS.

[113] Ficarra M., Ibana J.S.A., Poretta C., Ma L., Myers L., Taylor S.N., Greene S., Smith B., Hagensee M., Martin D.H. (2008). A Distinct Cellular Profile Is Seen in the Human Endocervix during Chlamydia trachomatis Infection. Am. J. Reprod. Immunol..

[114] McGowin C.L., Popov V.L., Pyles R.B. (2009). Intracellular Mycoplasma genitalium infection of human vaginal and cervical epithelial cells elicits distinct patterns of inflammatory cytokine secretion and provides a possible survival niche against macrophage-mediated killing. BMC Microbiol..

[115] Vieira V.A., Avelino-Silva V.I., Cerqueira N.B., Costa D.A., Costa P.R., Vasconcelos R.P., Madruga V.R., Moreira R.I., Hoagland B., Veloso V.G. (2017). Asymptomatic anorectal Chlamydia trachomatis and Neisseria gonorrhoeae infections are associated with systemic CD8+ T-cell activation. AIDS.

[116] Cohen M.S., Hoffman I.F., A Royce R., Kazembe P., Dyer J.R., Daly C.C., Zimba D., Vernazza P.L., Maida M., A Fiscus S. (1997). Reduction of concentration of HIV-1 in semen after treatment of urethritis: Implications for prevention of sexual transmission of HIV-1. AIDSCAP Malawi Research Group. Lancet.

[117] Fu J., E Sha B., Thomas L.L. (2011). HIV-1–Infected Peripheral Blood Mononuclear Cells Enhance Neutrophil Survival and HLA-DR Expression via Increased Production of GM-CSF: Implications for HIV-1 Infection. Am. J. Ther..

[118] Dzakah E.E., Zhao J., Wang L., Rashid F., Xu R., Yang L., Wan Z., Huang L., Wang H., Chen S. (2021). Chlamydia trachomatis Stimulation Enhances HIV-1 Susceptibility through the Modulation of a Member of the Macrophage Inflammatory Proteins. J. Investig. Dermatol..

[119] Gheit T. (2019). Mucosal and Cutaneous Human Papillomavirus Infections and Cancer Biology. Front. Oncol..

[120] Kombe A.J.K., Li B., Zahid A., Mengist H.M., Bounda G.-A., Zhou Y., Jin T. (2021). Epidemiology and Burden of Human Papillomavirus and Related Diseases, Molecular Pathogenesis, and Vaccine Evaluation. Front. Public Health.

[121] Schiffman M., Doorbar J., Wentzensen N., de Sanjosé S., Fakhry C., Monk B.J., Stanley M.A., Franceschi S. (2016). Carcinogenic human papillomavirus infection. Nat. Rev. Dis. Prim..

[122] Longworth M.S., Laimins L.A. (2004). Pathogenesis of Human Papillomaviruses in Differentiating Epithelia. Microbiol. Mol. Biol. Rev..

[123] Zhou C., Tuong Z.K., Frazer I.H. (2019). Papillomavirus Immune Evasion Strategies Target the Infected Cell and the Local Immune System. Front. Oncol..

[124] Steinbach A., Riemer A.B. (2017). Immune evasion mechanisms of human papillomavirus: An update. Int. J. Cancer.

[125] Westrich J.A., Warren C.J., Pyeon D. (2017). Evasion of host immune defenses by human papillomavirus. Virus Res..

[126] Doorbar J., Quint W., Banks L., Bravo I.G., Stoler M., Broker T.R., Stanley M.A. (2012). The Biology and Life-Cycle of Human Papillomaviruses. Vaccine.

[127] Kilic C. (2022). Association of Human Papillomavirus and Chlamydia trachomatis Coinfection with Cervical Intraepithelial Lesions and Cervical Cancer. Curr. Obstet. Gynecol. Rep..

[128] Chen H., Luo L., Wen Y., He B., Ling H., Shui J., He P., Hou X., Tang S., Li Z. (2020). Chlamydia trachomatis and Human Papillomavirus Infection in Women from Southern Hunan Province in China: A Large Observational Study. Front. Microbiol..

[129] Nonato D.R., Alves R.R., Ribeiro A.A., Saddi V.A., Segati K.D., Almeida K.P., de Lima Y.A., D’alessandro W.B., Rabelo-Santos S.H. (2016). Prevalence and factors associated with coinfection of human papillomavirus and Chlamydia trachomatis in adolescents and young women. Am. J. Obstet. Gynecol..

[130] Verteramo R., Pierangeli A., Mancini E., Calzolari E., Bucci M., Osborn J., Nicosia R., Chiarini F., Antonelli G., Degener A.M. (2009). Human Papillomaviruses and genital co-infections in gynaecological outpatients. BMC Infect. Dis..

[131] Olivera C., Mosmann J.P., Paira D.A., Molina R.I., Tissera A.D., Motrich R.D., Cuffini C.G., Rivero V.E. (2021). Association between Human Papillomavirus and Chlamydia trachomatis genital infections in male partners of infertile couples. Sci. Rep..

[132] Pérez-Soto E., Fernández-Martínez E., Oros-Pantoja R., Medel-Flores O., Miranda-Covarrubias J.C., Sánchez-Monroy V. (2021). Proinflammatory and Oxidative Stress States Induced by Human Papillomavirus and *Chlamydia trachomatis* Coinfection Affect Sperm Quality in Asymptomatic Infertile Men. Medicina.

[133] Naldini G., Grisci C., Chiavarini M., Fabiani R. (2019). Association between human papillomavirus and chlamydia trachomatis infection risk in women: A systematic review and meta-analysis. Int. J. Public Health.

[134] Tamim H., Finan R.R., Sharida H.E., Rashid M., Almawi W.Y. (2002). Cervicovaginal coinfections with human papillomavirus and chlamydia trachomatis. Diagn. Microbiol. Infect. Dis..

[135] Yang D., Zhang J., Cui X., Ma J., Wang C., Piao H. (2022). Risk Factors Associated with Human Papillomavirus Infection, Cervical Cancer, and Precancerous Lesions in Large-Scale Population Screening. Front. Microbiol..

[136] Chelimo C., Wouldes T.A., Cameron L.D., Elwood J.M. (2013). Risk factors for and prevention of human papillomaviruses (HPV), genital warts and cervical cancer. J. Infect..

[137] Bébéar C., de Barbeyrac B. (2009). Genital Chlamydia trachomatis infections. Clin. Microbiol. Infect..

[138] Menon S., Timms P., Allan J.A., Alexander K., Rombauts L., Horner P., Keltz M., Hocking J., Huston W.M. (2015). Human and Pathogen Factors Associated with Chlamydia trachomatis-Related Infertility in Women. Clin. Microbiol. Rev..

[139] Huai P., Li F., Li Z., Sun L., Fu X., Pan Q., Yu G., Chai Z., Chu T., Mi Z. (2018). Prevalence, risk factors, and medical costs of Chlamydia trachomatis infections in Shandong Province, China: A population-based, cross-sectional study. BMC Infect. Dis..

[140] Ji Y., Ma X.-X., Li Z., Peppelenbosch M.P., Ma Z., Pan Q. (2018). The Burden of Human Papillomavirus and *Chlamydia trachomatis* Coinfection in Women: A Large Cohort Study in Inner Mongolia, China. J. Infect. Dis..

[141] Samoff E., Koumans E.H., Markowitz L.E., Sternberg M., Sawyer M.K., Swan D., Papp J.R., Black C.M., Unger E.R. (2005). Association of Chlamydia trachomatis with Persistence of High-Risk Types of Human Papillomavirus in a Cohort of Female Adolescents. Am. J. Epidemiol..

[142] Lugo L.Z.A., Puga M.A.M., Jacob C.M.B., Padovani C.T.J., Nocetti M.C., Tupiná M.S., Pina A.F.S., de Freitas J.N.M., Ferreira A.M.T., Fernandes C.E.d.S. (2023). Cytokine profiling of samples positive for Chlamydia trachomatis and Human papillomavirus. PLoS ONE.

[143] Simonetti A.C., Melo J.H.d.L., de Souza P.R.E., Bruneska D., Filho J.L.d.L. (2009). Immunological’s host profile for HPV and Chlamydia trachomatis, a cervical cancer cofactor. Microbes Infect..

[144] Wallin K.-L., Wiklund F., Luostarinen T., Ångström T., Anttila T., Bergman F., Hallmans G., Ikäheimo I., Koskela P., Lehtinen M. (2002). A population-based prospective study of *Chlamydia trachomatis* infection and cervical carcinoma. Int. J. Cancer.

[145] Scurtu L.G., Jinga V., Simionescu O. (2022). Fascinating Molecular and Immune Escape Mechanisms in the Treatment of STIs (Syphilis, Gonorrhea, Chlamydia, and Herpes Simplex). Int. J. Mol. Sci..

[146] Discacciati M.G., Gimenes F., Pennacchi P.C., Faião-Flores F., Zeferino L.C., Derchain S.M., Teixeira J.C., Costa M.C., Zonta M., Termini L. (2015). MMP-9/RECK Imbalance: A Mechanism Associated with High-Grade Cervical Lesions and Genital Infection by Human Papillomavirus and *Chlamydia trachomatis*. Cancer Epidemiol. Biomark. Prev..

[147] Yang X., Siddique A., Khan A.A., Wang Q., Malik A., Jan A.T., Rudayni H.A., Chaudhary A.A., Khan S. (2021). *Chlamydia Trachomatis* Infection: Their potential implication in the Etiology of Cervical Cancer. J. Cancer.

[148] DeFilippis R.A., Goodwin E.C., Wu L., DiMaio D. (2003). Endogenous Human Papillomavirus E6 and E7 Proteins Differentially Regulate Proliferation, Senescence, and Apoptosis in HeLa Cervical Carcinoma Cells. J. Virol..

[149] Franchini A.P.A., Iskander B., Anwer F., Oliveri F., Fotios K., Panday P., Hamid P. (2022). The Role of Chlamydia Trachomatis in the Pathogenesis of Cervical Cancer. Cureus.

[150] Wang K., Muñoz K.J., Tan M., Sütterlin C. (2021). *Chlamydia* and HPV induce centrosome amplification in the host cell through additive mechanisms. Cell. Microbiol..

[151] Mirzayans R., Andrais B., Murray D. (2018). Roles of Polyploid/Multinucleated Giant Cancer Cells in Metastasis and Disease Relapse Following Anticancer Treatment. Cancers.

[152] Knowlton A.E., Fowler L.J., Patel R.K., Wallet S.M., Grieshaber S.S. (2013). Chlamydia Induces Anchorage Independence in 3T3 Cells and Detrimental Cytological Defects in an Infection Model. PLoS ONE.

[153] Bhatla N., Puri K., Joseph E., Kriplani A., Iyer V.K., Sreenivas V. (2013). Association of Chlamydia trachomatis infection with human papillomavirus (HPV) & cervical intraepithelial neoplasia—A pilot study. Indian J. Med. Res..

[154] Mosmann J.P., Zayas S., Kiguen A.X., Venezuela R.F., Rosato O., Cuffini C.G. (2021). Human papillomavirus and Chlamydia trachomatis in oral and genital mucosa of women with normal and abnormal cervical cytology. BMC Infect. Dis..

[155] Quinn R., Salvatierra J., Solari V., Calderon M., Ton T.G., Zunt J.R., Müller E.E., Rebe K., Chirwa T.F., Struthers H. (2012). Human Papillomavirus Infection in Men Who Have Sex with Men in Lima, Peru. AIDS Res. Hum. Retrovir..

[156] Agelidis A.M., Shukla D., Isa P., Gutiérrez M., Arias C.F., López S., Tang H., Mori Y., Ogden S.C., Tang H. (2015). Cell entry mechanisms of HSV: What we have learned in recent years. Futur. Virol..

[157] Fatahzadeh M., Schwartz R.A. (2007). Human herpes simplex virus infections: Epidemiology, pathogenesis, symptomatology, diagnosis, and management. J. Am. Acad. Dermatol..

[158] (WHO) World Health Organization (2023). Herpes Simplex Virus.

[159] Looker K.J., Welton N.J., Sabin K.M., Dalal S., Vickerman P., Turner K.M.E., Boily M.-C., Gottlieb S.L. (2020). Global and regional estimates of the contribution of herpes simplex virus type 2 infection to HIV incidence: A population attributable fraction analysis using published epidemiological data. Lancet Infect. Dis..

[160] A Lamb C., Lamb E.I.M., Mansfield J.C., Sankar K.N. (2012). Sexually transmitted infections manifesting as proctitis. Front. Gastroenterol..

[161] Margolis T.P., Imai Y., Yang L., Vallas V., Krause P.R. (2007). Herpes Simplex Virus Type 2 (HSV-2) Establishes Latent Infection in a Different Population of Ganglionic Neurons than HSV-1: Role of Latency-Associated Transcripts. J. Virol..

[162] Retamal-Díaz A.R., Tognarelli E., Kalergis A.M., Bueno S.M., González P.A. (2016). Immune Evasion by Herpes Simplex Viruses. Herpesviridae.

[163] Melchjorsen J., Matikainen S., Paludan S.R. (2009). Activation and Evasion of Innate Antiviral Immunity by Herpes Simplex Virus. Viruses.

[164] White D.W., Beard R.S., Barton E.S. (2011). Immune modulation during latent herpesvirus infection. Immunol. Rev..

[165] Mantell J., Goh B.T., Woodland R.M., Walpita P. (1988). Dual Infection of the Conjunctiva with Herpes Simplex Virus and Chlamydia trachomatis. Sex. Transm. Dis..

[166] Mosmann J.P., Talavera A.D., Criscuolo M.I., Venezuela R.F., Kiguen A.X., Panico R., De Prato R.F., De Blanc S.A.L., Ré V., Cuffini C.G. (2019). Sexually transmitted infections in oral cavity lesions: Human papillomavirus, *Chlamydia trachomatis*, and Herpes simplex virus. J. Oral Microbiol..

[167] Vahidnia A., Buijs I.O.D., Roymans R., Bliekendaal H., van de Bovenkamp J. (2013). A retrospective study into the prevalence of herpes simplex virus 1&2 in female patients tested for Chlamydia trachomatis and/or Neisseria gonorrhoeae using vaginal swabs. Clin. Microbiol. Infect..

[168] Shaw S.Y., Deering K.N., Reza-Paul S., Isac S., Ramesh B.M., Washington R., Moses S., Blanchard J.F. (2011). Prevalence of HIV and sexually transmitted infections among clients of female sex workers in Karnataka, India: A cross-sectional study. BMC Public Health.

[169] Paroli E., Franco E., Mele A., Caprilli F., Gentili G., Stazi M.A., Corona R., Felici F., Prignano G., Palamara G. (1990). Seroprevalence of anti-Chlamydia trachomatis IgG in outpatients attending a sexually transmitted disease clinic in Italy. Eur. J. Epidemiol..

[170] Vetter K.M., Barnes R.C., Oberle M.W., Rosero-Bixby L., Schachter J. (1990). Seroepidemiology of chlamydia in Costa Rica. Sex. Transm. Infect..

[171] Slade J., Hall J.V., Kintner J., Schoborg R.V. (2016). Chlamydial Pre-Infection Protects from Subsequent Herpes Simplex Virus-2 Challenge in a Murine Vaginal Super-Infection Model. PLoS ONE.

[172] Kalimo K., Terho P., Honkonen E., Gronroos M., Halonen P. (1981). Chlamydia trachomatis and herpes simplex virus IgA antibodies in cervical secretions of patients with cervical atypia. BJOG: Int. J. Obstet. Gynaecol..

[173] Maral I., Biri A., Korucuoğlu Ü., Bakar C., Çırak M., Bumin M.A. (2009). Seroprevalences of herpes simplex virus type 2 and Chlamydia trachomatis in Turkey. Arch. Gynecol. Obstet..

[174] Rahman S., Wathington D., Waterboer T., Pawlita M., Villa L.L., Lazcano-Ponce E., Willhauck-Fleckenstein M., Brenner N., Giuliano A.R. (2021). Seroprevalence of Chlamydia trachomatis, herpes simplex 2, Epstein-Barr virus, hepatitis C and associated factors among a cohort of men ages 18–70 years from three countries. PLoS ONE.

[175] Duru C.B., Emele F.E., Nnebue C.C., Adinma E.D., Ifeadike G.O., Amilo G.I., Oluboyo A.O., Oluboyo B.O. (2014). The Seroprevalence and Co-Existence of Chlamydia Trachomatis and Herpes Simplex Virus Antibodies among Students in a Tertiary Institution. Int. J. Med. Med. Sci..

[176] Kajaia D., Merabishvili N., Burkadze G. (2006). Pap testing and direct immunofluorescence for Chlamydia trachomatis infection in pregnant women. Georgian Med. News.

[177] Chiarini F., Mansi A., Pisani S., Seganti L., Brunori S., Gentile V., Di Silverio F. (1996). In vitro study of a double infection by herpes simplex virus type 2 and Chlamydia trachomatis. New Microbiol..

[178] Domke-Opitz I., Straub P., Kirchner H. (1986). Effect of interferon on replication of herpes simplex virus types 1 and 2 in human macrophages. J. Virol..

[179] Bochud P., Magaret A.S., Koelle D.M., Aderem A., Wald A. (2007). Polymorphisms in *TLR2* Are Associated with Increased Viral Shedding and Lesional Rate in Patients with Genital Herpes Simplex Virus Type 2 Infection. J. Infect. Dis..

[180] Yao X.-D., Rosenthal K.L. (2011). Herpes simplex virus type 2 virion host shutoff protein suppresses innate dsRNA antiviral pathways in human vaginal epithelial cells. J. Gen. Virol..

[181] Vanover J., Kintner J., Whittimore J., Schoborg R.V. (2010). Interaction of herpes simplex virus type 2 (HSV-2) glycoprotein D with the host cell surface is sufficient to induce Chlamydia trachomatis persistence. Microbiology.

[182] Musarrat F., Jambunathan N., Rider P.J.F., Chouljenko V.N., Kousoulas K.G. (2018). The Amino Terminus of Herpes Simplex Virus 1 Glycoprotein K (gK) Is Required for gB Binding to Akt, Release of Intracellular Calcium, and Fusion of the Viral Envelope with Plasma Membranes. J. Virol..

[183] Hall J.V., Sun J., Slade J., Kintner J., Bambino M., Whittimore J., Schoborg R.V. (2014). Host nectin-1 is required for efficient *Chlamydia trachomatis* serovar E development. Front. Cell. Infect. Microbiol..

[184] Fierz W. (1999). Basic Problems of Serological Laboratory Diagnosis. Mol. Biotechnol..

[185] Murray P.R. (2015). The Clinician and the Microbiology Laboratory. Mandell, Douglas, and Bennett’s Principles and Practice of Infec-tious Diseases.

